# Fabrication Methods of Electroactive Scaffold-Based Conducting Polymers for Tissue Engineering Application: A Review

**DOI:** 10.3389/fbioe.2022.876696

**Published:** 2022-07-07

**Authors:** Nurul Ain Najihah Asri, Mohd Muzamir Mahat, Azlan Zakaria, Muhd Fauzi Safian, Umi Marshida Abd Hamid

**Affiliations:** ^1^ School of Physics and Material Studies, Faculty of Applied Sciences, Universiti Teknologi MARA, Shah Alam, Malaysia; ^2^ School of Industrial Technology, Faculty of Applied Sciences, Universiti Teknologi MARA, Shah Alam, Malaysia; ^3^ School of Chemistry and Environmental Studies, Faculty of Applied Sciences, Universiti Teknologi MARA, Shah Alam, Malaysia; ^4^ School of Biology, Faculty of Applied Sciences, Universiti Teknologi MARA, Shah Alam, Malaysia

**Keywords:** conducting polymer, tissue engineering, conventional method, rapid prototyping, electrospinning, 3D printing, bioprinting, 4D printing

## Abstract

Conductive scaffolds, defined as scaffold systems capable of carrying electric current, have been extensively researched for tissue engineering applications. Conducting polymers (CPs) as components of conductive scaffolds was introduced to improve morphology or cell attachment, conductivity, tissue growth, and healing rate, all of which are beneficial for cardiac, muscle, nerve, and bone tissue management. Conductive scaffolds have become an alternative for tissue replacement, and repair, as well as to compensate for the global organ shortage for transplantation. Previous researchers have presented a wide range of fabrication methods for conductive scaffolds. This review highlights the most recent advances in developing conductive scaffolds, with the aim to trigger more theoretical and experimental work to address the challenges and prospects of these new fabrication techniques in medical sciences.

## 1 Introduction

Tissue and organ failure resulting from injuries, diseases, or congenital disabilities is currently one of the most severe public health concerns, with increasing incidences worldwide. According to statistics, in the United States, one person waits for an organ transplant every 15 minutes. Unfortunately, due to the exponential growth in the expectant list, less than half of the waiting patients are fortunate to obtain a suitable organ from a pardoner (Saberi et al., 2019). Furthermore, patients are at risk of donor tissue morbidity, infectious diseases, and pain. Similarly, Massoumi et al. (2019) reported that it was well established for a neuronal tissue not to regenerate once damaged because it lacked stem cells and thus would not self-regenerate. As a result, tissue engineering (TE) has received increased attention and has emerged as a powerful alternative in the biomedical field for saving lives and improving quality of life.

Tissue engineering, alternatively referred to as regenerative medicine, is a multidisciplinary and interdisciplinary field that makes use of engineering principles and life sciences principles to create functional biological substitutes for native tissues that restore, maintain, improve, or replace biological functions by combining a scaffold, cells, and biological molecules without the use of organ transplantation (Guo & Ma, 2018; Sultana et al., 2020). Furthermore, TE techniques have been widely used on various tissues and organs, including the heart, skin, muscle, nerve, bone, cartilage, and cornea (Saberi et al., 2019). TE techniques should begin with a scaffold to create an environment for cells or tissues to develop in an organized manner before establishing new tissues or organs (Jun et al., 2018). Therefore, artificial scaffolds are currently being used as a supporting system to heal damaged tissues or organs for cell culture and growth.

Scaffolds are made of a range of synthetic or natural polymers which provide structural support and three-dimensional template for tissue regeneration (Dhandayuthapani et al., 2011; Ramburrun et al., 2019). Furthermore, Boffito et al. (2014) has asserted that scaffold can be utilized into two different circumstances: 1) As *in vivo* regeneration by providing cell structure supports and function restoring through cell recruitment from surrounding tissues, and 2) as *ex vivo* and *in vivo* regeneration of a new tissue from seeded cells. There are several types of scaffolds available for clinical use such as, porous scaffold (Nilghaz et al., 2018), hydrogel scaffold (Navaei et al., 2019; Ahmad Ruzaidi et al., 2021), microsphere scaffold (Jaklenec et al., 2008; Patel et al., 2021), and fibrous scaffold (Campbell et al., 2021).

Porous scaffolds with appropriate and sufficient porosity of suitable size and interconnection are essential for the porous tissue architecture application, such as bone tissue engineering, which subsequently creates an environment that promotes cell infiltration, migration, vascularization, nutrient and oxygen flow, and waste disposal while enduring external loading loads (Cheng et al., 2019; Abbasi et al., 2020). Nonetheless, hydrogels have evolved as among the most prominent and diverse groups of materials utilized in tissue engineering due to the nature of hydrogels that attract and retain water molecules (Jeong et al., 2017). Consequently, hydrogels can be designed to mimic native soft tissues due to their highly hydrated environment with a water content of ≥90% by weight (Jeong et al., 2017; Naahidi et al., 2017; Spicer, 2020). Moreover, microsphere scaffolds have been extensively employed in drug delivery due to its potentiality to enhance the efficacy of encapsulated drug by providing large surface area–to–volume ratio and spatial and temporal control over release of bioactive molecules for tissue regeneration (Gupta et al., 2017). Another type of scaffold is fibrous scaffold. Fibrous scaffold can be developed in nano- or microscale fibrous structure with interconnected pores that resemble extracellular matrix (ECM) of the native tissues while possess great ability to facilitate the development of artificial functional tissues (Jun et al., 2018). Table 1 shows the different types of scaffolds and their findings.

**TABLE 1:** Summary on the different types of scaffolds and their findings.

Scaffold type	Major findings	References
Porous scaffold	A three-dimensional (3D) cell culture system was fabricated by stacking four layers of polydimethylsiloxane (PDMS) supported by thread and embedded with functionalized hydroxypropyl cellulose methacrylate (HPC-MA) porous scaffold. The sewn thread was located into the PDMS channel for media transportation to the cells in scaffold and waste discharge from the scaffold construct. In single thread scaffold system, COS-7 cells proliferated on Day 3, however, unable to survive until Day 6 due to the delivered nutrients scarcity and inability of waste removal from the scaffold construct. Hence, supplemental cotton threads were positioned to each PDMS layer after Day 3 for nutrients sufficiency to the cells present in the scaffold.	Nilghaz et al. (2018)
Hydrogel scaffold	An electroconductive chitosan/gelatin/agar based PEDOT: PSS hydrogel was developed *via* thermal reverse casting method. The hydrogel contained 1% v/v of DMSO-doped PEDOT: PSS demonstrated an optimum conductivity value of 3.35 × 10^−4^ S/cm. As the volume of doped PEDOT: PSS content increased to 1.5% v/v, there was a gradual decrease in conductivity value to 3.28 × 10^−4^ S/cm. Furthermore, all of the hydrogel samples (n = 5) showed no significant difference in terms of color intensity after being submerged for 30 min in phosphate buffer solution (PBS) for stability testing.	Ahmad Ruzaidi et al. (2021)
Microsphere scaffold	An alternative bone graft substitution was fabricated by utilizing alginate-graphene oxide-dexamethasone (Alg-GO-Dex) composite microspheres through calcium ion crosslinking, followed by air dry and freeze-drying method. The synthesized microspheres had a porosity of more than 80% and homogenous GO dispersion in the alginate matrix. These GO dispersion to the alginate matrix improved drug encapsulation efficiency by improving MG-63 cell adhesion and proliferation. Also, the composite microspheres provide excellent sustained drug release, *in vitro* biomineralization, and biocompatible. The inclusion of dexamethasone in the microsphere system stimulated cell proliferation and boosted apatite formation.	Yashaswini et al. (2021)
Fibrous scaffold	An engineered resveratrol-loaded fibrous scaffolds were fabricated *via* electrospinning method. The polycaprolactone embedded resveratrol (PCL-R) scaffolds demonstrated decreased inflammatory cell infiltration, improved collagen ECM secretion, and blood vessel network formation following myocardial infarction (MI). Also, the immunofluorescence analysis disclosed resveratrol-loaded scaffolds promote increased expression of cTnT, Cx-43, Trx-1, and VEGF proteins.	Campbell et al. (2021)

**FIGURE 1:**
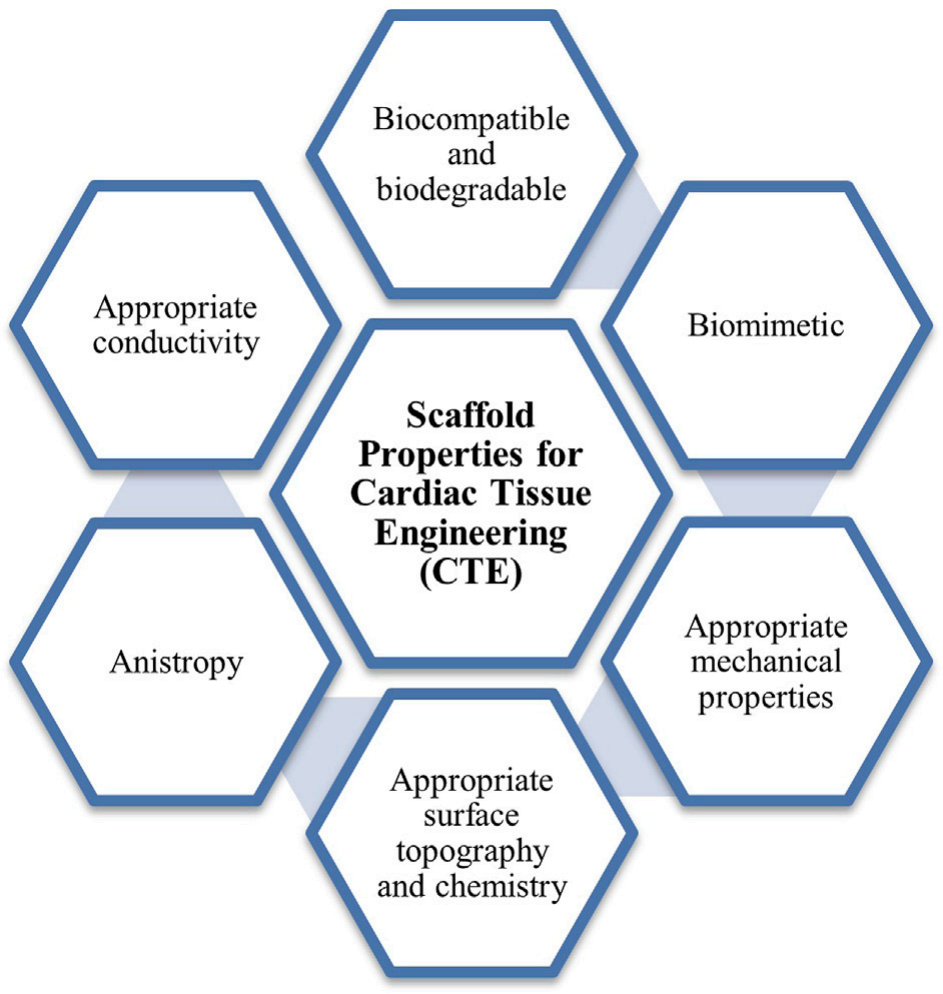
Scaffold properties required for CTE.

Primarily, the scaffold must possess good biocompatibility, biodegradability, and biomimicry. According to the Williams definition of biocompatibility, biocompatible defines as the ability of a material to operate with an adequate host response in a specific application, depending on their cytocompatibility, pathogenicity, immunogenicity, and biodegradability of decellularized tissues and organs (Hussein et al., 2016). Additionally, the scaffolds must gradually and naturally degrade into a non-toxic degradation products during or after the healing process. Also, the scaffold should mimic the native cardiac extracellular matrix (ECM) properties in terms of the geometrical and anisotropic structure, mechanical and topography properties. In addition, conductivity is an essential criterion for scaffold-assisted excitable tissue regeneration, including cardiac, skeletal, and smooth muscles, as well as neural tissues (Sikorski, 2020). Therefore, conducting polymer (CP) scaffolds were developed to meet the requirements of electroconductive scaffolds by incorporating the softness of polymeric materials with the electrical properties of conducting polymers (CPs) (Jayaram et al., 2019). This review focuses on revealing the significance of incorporating intrinsically conductive polymers into the scaffolds construct *via* recent advances fabrication methods which contributed significantly to geometrical and pores uniformity, cell proliferation, mechanical properties, and conductivity. The scaffold properties required for cardiac tissue engineering (CTE) were elucidated in Figure 1.

## 2 Current Fabrication Technologies of Scaffolds

**FIGURE 2:**
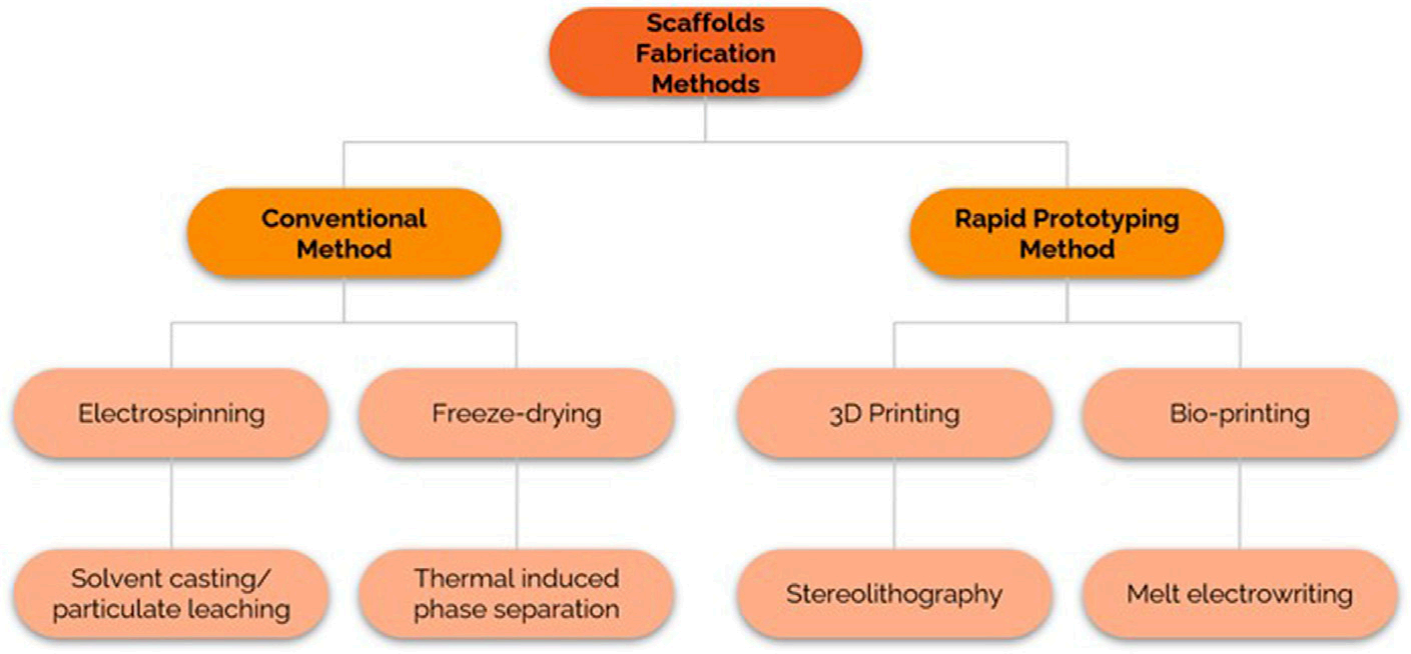
Scaffold fabrication methods comprised of conventional and rapid prototyping methods.

Scaffold fabrication techniques are divided into traditional and modern/rapid prototyping (RP) methods (Eltom et al., 2019), as illustrated in Figure 2. By constructing porous polymer structures with the goal of cell adhesion, the traditional technique has demonstrated tremendous assurance in scaffold fabrication. Electrospinning (Shamsah et al., 2020), freeze-drying (Janik & Marzec, 2015), solvent casting/particulate leaching (SCPL) (Prasad et al., 2017), and thermally induced phase separation (TIPS) are some examples of conventional methods (Conoscenti et al., 2017). These conventional methods can create scaffolds with high interconnectivity and porosity and homogeneous pore size, mimicking extracellular matrix (ECM) (Peng et al., 2018). However, traditional approaches make it difficult to construct complex structures with tuneable micro- and macroscales.

As a result, rapid prototyping (RP) technology emerges as a series of layer-by-layer additive manufacturing process to construct complex three-dimensional (3D) architecture that can eventually be tailored to accommodate patient-specific applications (Hoque et al., 2011). RP allows for the control of scaffold pore size by converting computer data obtained from Computer-Aided Design (CAD), Computer Tomography (CT), and Magnetic Resonance Imaging (MRI) analyses (Liu et al., 2017; Touri et al., 2019). Three types of RP systems exist based on the initial form of the feed materials: liquid-based, solid-based, and powder-based (Touri et al., 2019). RP technology includes 3D printing (Lei et al., 2019), bioprinting (Rider et al., 2018), stereolithography (SLA) (Guillaume et al., 2017) and melt electro-writing (MEW) (Castilho et al., 2018).

**FIGURE 3:**
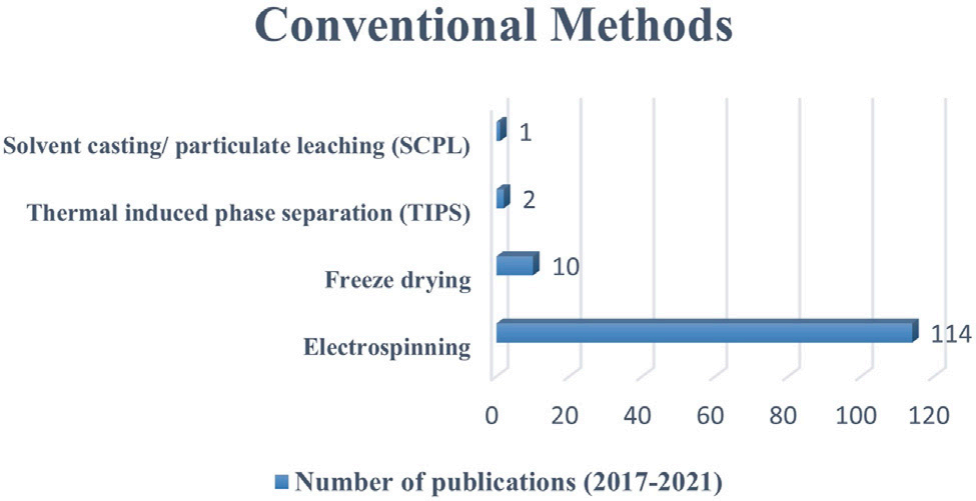
The number of publications on the use of conventional methods to fabricate conductive scaffolds between 2017 to 2021 (five-year period). Data as of 1st September 2021 from Web of Science.

**FIGURE 4:**
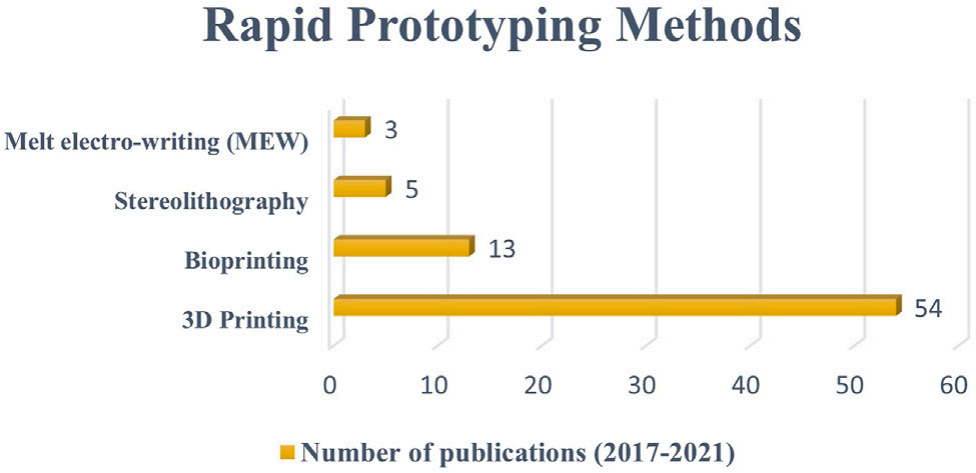
The number of publications on the use of rapid prototyping methods to fabricate conductive sscaffolds between 2017 to 2021 (five-year period). Data as of 1st September 2021 from Web of Science.

This review presents the most used scaffold fabrication methods to produce conductive scaffolds for tissue engineering purposes reported within the last 5 years. The conductive scaffolds fabrication which involved CPs as the component has shown no major impact on their processing (Abedi et al., 2019; Wibowo et al., 2020). This is due to the similar nature of polymer and the scaffold-based materials. However, if the conductive components are metal-based, the application process would be different which affected by their homogeneity and miscibility (Maharjan et al., 2017; Angulo-pineda et al., 2020). Primarily, the electrospinning method received the most publications, indicating that it was the most widely used scale method. Subsequently, freeze-drying, thermal induced phase separation, and solvent casting/particulate leaching methods recorded lower number of methods used (Figure 3). In terms of RP methods, 3D printing received the most publications, followed by bioprinting, stereolithography, and melt electro-writing (Figure 4). These data on the number of publications were obtained from Web of Science, based on a search term of ‘name of the fabrication technique-, ‘tissue engineering conductive scaffold’.

## 3 Conventional Methods

Many conventional methods, such as electrospinning, freeze-drying, TIPS, and SCPL have been investigated for the development of TE scaffolds. However, as previously stated, electrospinning has emerged as the most popular method for developing TE scaffolds, such as conductive nanofibrous scaffolds containing PEDOT: PSS targeted for cardiac tissue engineering (CTE) and neural tissue, and electrically conductive electrospun silk scaffolds for electrically sensitive tissues.

### 3.1 Electrospinning

In the 19th century, John William Strutt (Lord Rayleigh) observed the phenomenon of electrospinning for the first time. Charles Vernon Boys designed and built the set-up primarily with molten waxes as they drew fibers. John Francis Cooley and William James Morton later filed the first industrial electrospinning patents in 1900 and 1902. Electrospinning was founded on universal agreement in 1934, when Anton Formhals began patenting numerous electrospinning techniques. After nearly 10 years and 22 patents, Formhals significantly improved the procedure and established electrospinning as a viable and efficient approach. Sir Geoffrey Ingram Taylor introduced the principles theory of electrospinning a few years later, in the 1960s, focusing on the jet forming process.

Electrospinning, which derives from the term “electrostatic spinning,” is a spinning technique that employs electrostatic forces to produce electrospun fibers with diameters ranging from micrometers to nanometers depending on the polymer types and processing conditions (Jun et al., 2018; Torabi et al., 2016). Furthermore, electrospinning is an appealing method for producing polymer biomaterials because it allows for simple equipment to monitor morphology, porosity, and composition (Bera, 2016). For several decades, electrospinning has been used in the tissue engineering field to fabricate ECM-mimicking fibrous scaffolds from biocompatible polymers (Jun et al., 2018).

**FIGURE 5:**
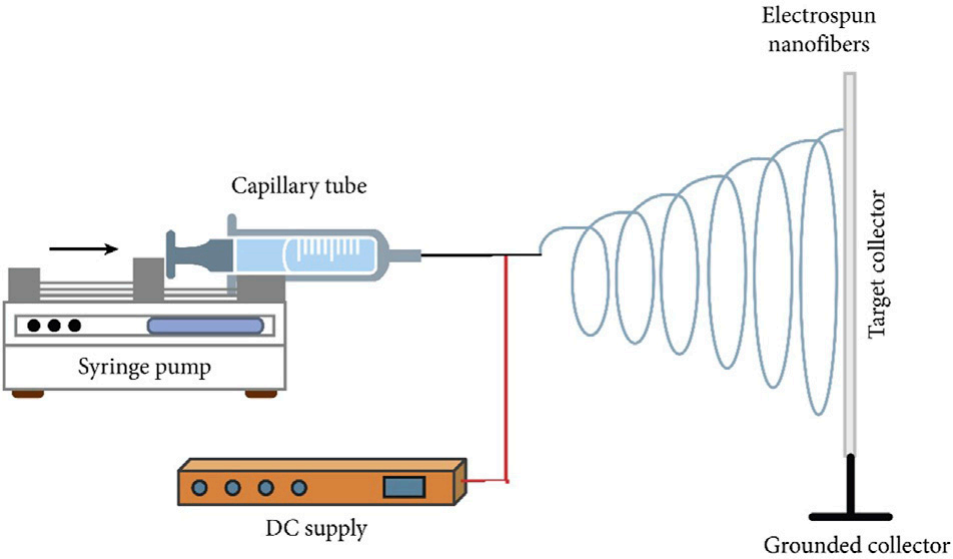
Schematic illustration of the electrospinning set-up. Image was adapted from Eltom et al. (2019) under the Creative Commons Attribution License.

In general, the electrospinning process requires four main components: a glass syringe containing a polymer solution, a metallic needle, a high-voltage power supply, and a metallic collector (Figure 5). The process begins when electrical charges are introduced into the polymer solution through the metallic needle. The induction of charges on the polymer droplet results in instability or volatility within the polymer solution. As the electrical field increases, the spherical droplet deforms and takes on a conical shape. The conical jet shape is dubbed the “Taylor cone” due to spinneret droplet distortion when electrostatic forces exceed surface tension and deposit ultrafine nanofibers at an optimized distance from the metallic collector (Haider et al., 2018; Falco & Mallavia, 2020). A stable charge jet can be produced only if the polymer solution has sufficient cohesive force. The applied voltage is typically between 5 and 30 kV, allowing for the ejection of a liquid jet followed by solvent evaporation. The jet flies, leaving ultra-fine polymer fibers behind. Ultra-fine polymer fibers are produced using a grounded cathode-connected metallic collector.

Several studies have attempted to implement tissue engineering by regulating the electrospinning process parameters, including the parameters of the electrospinning process, such as the operation of the electric field (Şener et al., 2011; Haider et al., 2018; Lasprilla-Botero et al., 2018), flow rate (Zhang et al., 2005; Haider et al., 2018), needle-tip-to-collector distance (Hekmati et al., 2013; Haider et al., 2018) and diameter of the metallic needle (Haider et al., 2018; Abdallah et al., 2021; Coelho et al., 2022), and the solution parameters, for instance, the concentration (Bosworth & Downes, 2012; Hekmati et al., 2013; Lasprilla-Botero et al., 2018). In addition, the environmental factors, for instance, relative temperature and humidity may affect the formation of bead-free electrospun fibers (Bae et al., 2013; Haider et al., 2018). The effects of electrospinning parameters and the solution concentration towards the fiber formation has been summarized in Table 2.

**TABLE 2:** Summary on the effect of electrospinning parameter and the solution concentration towards the fiber formation.

Electrospinning Parameters	Effect on the Fiber Formation
Applied voltage	At low voltage, Coulombic forces are insufficient to overcome the polymer solution’s surface tension, resulting in solvent spray (droplets and beads). At higher voltage, the surface tension and viscoelastic forces are relatively balanced, hence, allowing the formation of stable and straight jetting which produces a narrow fibers (Lasprilla-Botero et al., 2018). Also, at this relatively high yet balanced applied voltage, the polymeric fibers junction is reduced, which leads to the formation of uniform fiber distribution (Şener et al., 2011). If the applied voltage is increased further, the Coulombic forces may exceed the viscoelastic forces, resulting in the breakdown of the charged jet during flight, resulting in uneven fiber creation (Lasprilla-Botero et al., 2018). In addition, at higher voltage, the size of Taylor cone decreases due to the rapid jetting velocity at a constant flow rate. An uprising of applied voltage exceeding the value of critical voltage (of specified polymer), will cause the formation of beaded nanofibers (Haider et al., 2018). The size of beads increased with the applied voltage (Şener et al., 2011).
Flow rate of the polymeric solution	Uniform and bead-free electrospun nanofibers are formed at certain value of critical flow rate which varies depending on the polymeric solution (Haider et al., 2018). The formation of beaded nanofibers could happen as the flow rate increases (beyond the critical value), along with the increasing in pore size and fiber diameter (Zhang et al., 2005; Haider et al., 2018). These phenomenon resulted from the insufficient drying time of the nanofiber jets while travelling from the needle tip to the metallic collector.
Tip-to-collector distance (TCD)	The distance between the needle tip and the metallic collector is proportional to the evaporation rate of the solvent. In obtaining a defect-free/bead-less electrospun nanofibers, the passage duration between the needle tip and collector should be sufficient for solvent evaporation process to take place (Hekmati et al., 2013). TCD influenced the nanoweb collection zone diameter and the nanofibers’ average diameter. The diameter of the nanoweb collection zone reduces as the TCD lowers, although the average diameter of nanofibers grows dramatically (Hekmati et al., 2013; Haider et al., 2018). However, there are some occurrence where the shift in the TCD does not affect the nanofibers’ morphology (Haider et al., 2018).
Diameter of metallic needle	A reduction in needle diameter caused the surface tension of the polymer jets to increase, and subsequently decelerates the jetting from reaching the collector plate (Coelho et al., 2022). Hence, this phenomenon requires longer jetting to reach the collector plate while improving the drying time. Also, smaller needle diameter produced thinner and bead-less nanofibers compared to a larger needle diameter due to the stretching and thinning of polymer jets by the electrostatic forces (Abdallah et al., 2021).
Concentration	The initial solutions concentration give variation of morphology and fiber dimension of the nanofibers. Bosworth & Downes (2012) revealed a beaded morphology among the poly (ε-caprolactone) (PCL) fiber integration at the concentrations of 5 and 7.5% w/v and the fibers appeared thinner in diameter. However, as the solutions concentration increased to 10% w/v, there were no beads observed *via* SEM micrograph and the bead-free fibers appeared wider in diameter compared to the previous two concentrations. Nevertheless, Lasprilla-Botero et al. (2018) revealed that the polymer concentration for polyimide (PI) nanofibers should be above 15 wt% to achieve a more uniform morphology that is bead-free and form a regular electrospun fibers. At 15 wt% of PI, the polymer solution system has exceeded the entanglement concentration (Ce) and embarked into a concentrated system which allow for a stable and better jetting (Lasprilla-Botero et al., 2018). This is as a consequence of good cohesion between the polymer chains in the solution, and its stronger macromolecular arrangement (Hekmati et al., 2013; Lasprilla-Botero et al., 2018).

Compared to the rapid prototyping techniques based on CAD, electrospinning is a low-cost and straightforward method that has been developed for use in biological laboratories. Neither specialized engineers nor infrastructure (e.g., clean-room facilities in the case of photolithography) are required. A bio-composite conductive nanofibrous scaffold containing chitosan (CS) and PEDOT: PSS was fabricated *via* electrospinning, designed primarily for CTE (Abedi et al., 2019). For 10 hours, the prepared solution was electrospun using a double nozzle electrospinning equipment with a flow rate of 0.5 ml/h, a collector speed of 2500 rpm, a voltage of 20 kV, and a nozzle-to-collector distance of 18 cm. The addition of PEDOT: PSS to chitosan scaffolds improves their mechanical and electrical conductivity, as well as their biocompatibility and cell viability. The results indicate that increasing the PEDOT: PSS component to one wt% leads in a 30–40% reduction in fiber diameter and a nearly 100-fold increase in electrical conductivity. Additionally, the scaffold containing one wt% PEDOT: PSS boosts the tensile strength by approximately 9 MPa when compared to the neat sample. In conclusion, their findings are analogous to the extracellular matrix of the native myocardium and potentially applicable to CTE.

Similarly, Babaie et al. (2020) used electrospinning to create conductive composite scaffolds with PEDOT and polyvinyl alcohol (PVA) to mimic the natural environment of neural tissue. Samples were electrospun using a dual-nozzle set-up at 0.4 ml/h flow rate, 1000 rpm speed of aluminium wrapped collector, 25 kV applied voltage, and 18 cm needle-to-drum distance after the electrospinning parameters were optimized. It was reported that PEDOT-containing scaffolds outperform pure PVA scaffolds in terms of physicochemical properties and cell viability. Furthermore, PVA scaffolds containing one wt% PEDOT can effectively improve the electrical conductivity of non-conductive polymers while also improving the topographic and morphological properties of the fibers. Finally, it was proposed that using PEDOT as a conductive component in the fabrication of neural tissue engineering scaffolds can help improve the physical properties of the scaffolds and improve stem cell neural differentiation (Babaie et al., 2020).

The electrospinning technique has also been employed to produce conductive electrospun silk scaffolds functionalized with PEDOT: PSS and DMSO-treated PEDOT: PSS (Magaz et al., 2020). Electroconductive scaffolds have shown enormous promise for electrically sensitive tissues like nerves and muscle (cardiac, skeletal, and smooth), which rely primarily on electrochemical modulation between or within cells. Scaffolds were electrospun using a single nozzle electrospinning apparatus with a flow rate of 0.8 ml/h directed toward a static collector, an applied voltage of 15 kV, a tip-to-collector distance of 10 cm, relative humidity of 25%, and a needle gauge size of 19 G. The PEDOT: PSS conductive scaffolds has demonstrated the ability to support cell adhesion, proliferation, and differentiation. Thus, based on the evidence, the electrospinning method has successfully mimicked the ECM structure of native neural tissue by providing scaffold fiber diameters ranging from several nanometers to micrometers, similar to the structure of fibrillary proteins. The FESEM micrograph revealed that the inter-fiber pores/voids become partially occluded as PEDOT: PSS concentration increases. In contrast to the previous study, DMSO treatment was used to increase the electrical conductivity of the scaffold even further. Furthermore, DMSO treatment increased surface roughness, affecting protein adsorption and cellular responses such as cell adhesion.

## 4 Rapid Prototyping Methods (RPM)

Rapid prototyping (RP), also known as solid free-form fabrication and additive manufacturing (AM) among industrial professionals, has become state-of-the-art for conductive scaffold fabrication in recent years. Because of its high precision, significant reproducibility, and controllable inner pore structure, RPMs have been introduced in tissue engineering (TE) (Lee et al., 2017). Furthermore, using imaging data and computer-aided design (CAD) models, RPM can be tailored to fulfill patients’ actual conditions or requirements to effectively apply scaffolds during surgery.

**FIGURE 6:**
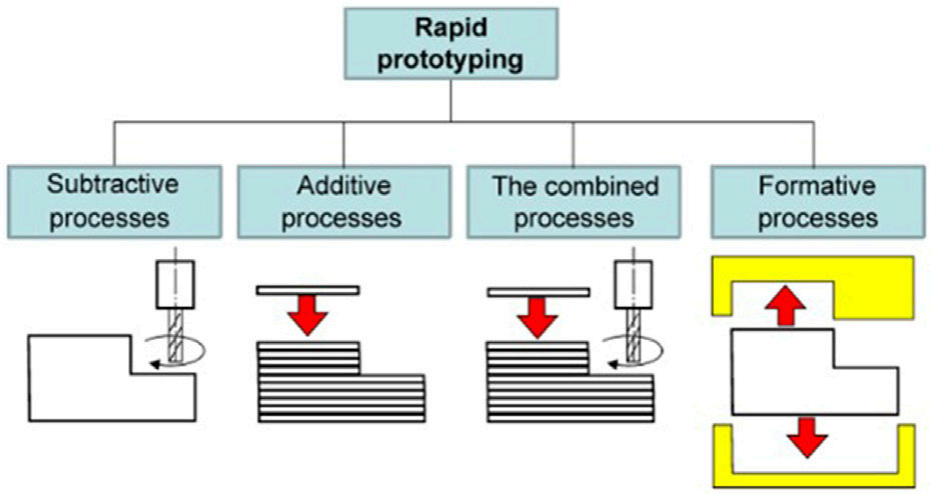
Fundamental classification of RPM. Image was adapted from Zivanovic et al. (2020) under the Creative Commons Attribution License.

According to Yuan and colleagues (2017), RPM consists of five steps, beginning with creating CAD models or collective physical entities using a digital method. The CAD model is then exported as a Stereolithographic (.STL) file for virtual slicing and digitally sliced into a cross-sectional layer as part of the pre-processing technique. In the fourth step, the RP system prints a single layer of the prototype simultaneously, while the workstation elevates or descends to the next layer until the entire process is completed. Finally, the hardening or surface treatment technique is dependent on the manufacturing technique and purpose. As illustrated in Figure 6, there are four fundamental RPM fabrication processes: 1) subtractive, 2) additive, (c) combined (subtractive and additive), and (d) formative (Ligon et al., 2017; Zivanovic et al., 2020).

### 4.1 3D Printing

Three-dimensional printing (3DP) is one of the most well-known RP technologies, developed more than 30 years ago by Charles Hull, who invented 3D lithography in 1986 (Su & Aref, 2018). The recent advancements of 3DP technologies; have increased the number of research using 3DP as a transformative tool for biomedical applications, particularly for tissue engineering and regenerative medicine (W. Zhu et al., 2016). Currently, 3DP is widely used to improve the applicability and functionality of cell-laden scaffolds and fabricate patient-specific scaffolds. It becomes a forerunner in the development of hierarchically advanced architectures that are not possible with current technology. Furthermore, 3DP creates objects by incorporating materials that minimize waste while achieving adequate geometric precision. It begins with a meshed 3D virtual model, which can be created with acquired image data or CAD models (X. Wang et al., 2017b).

Cox and colleagues (2015) previously demonstrated the use of 3DP to directly construct bone tissue scaffolds made of hydroxyapatite (HA) and polyvinyl alcohol (PVOH) composite precursor powders. Scaffolds with a porosity of 55% were created in Solidworks CAD software and saved in the standard ALM file format (.STL). The design was then printed on a ZPrinter 310+ 3D printer at 0.1 mm powder layer thickness and maximum binder saturation. A preliminary evaluation of this 3DP technique established 55% as the desired porosity threshold, which is ultimately encouraging because it aids in vascularization, fluid movement, and cell migration within the scaffold. Furthermore, the 3DP technique was chosen because 3D printed components have been shown to have good cell-biomaterial interaction due to the inherent roughness created by the imperfect packing of powdered stock materials. This phenomenon is advantageous for bone tissue engineering compared to other conventional methods, which are more likely to result in smooth extremities.

**FIGURE 7:**
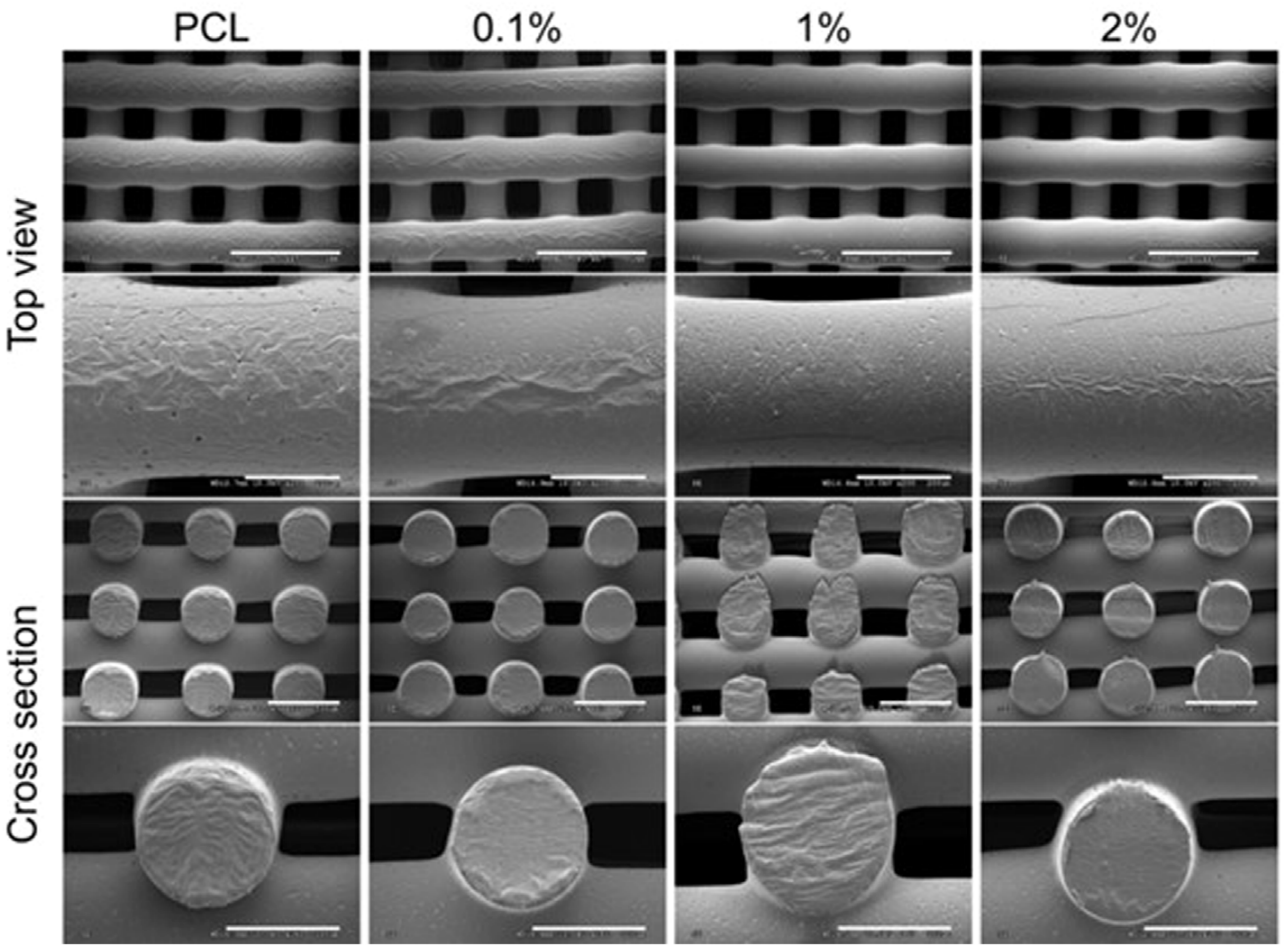
SEM images of PCL/ PANI scaffolds with varying PANI filler loading (0.1, 1, and 2% wt.). From top to bottom, the scale bars are 1 mm, 200 m, 500 m, and 300 m. Image was adapted from Wibowo et al. (2020) under the Creative Commons Attribution License.

Wibowo et al. (2020) recently developed a novel 3D-printed electroactive composite scaffold made of polycaprolactone (PCL) and polyaniline (PANI) for bone tissue applications. PANI at various weight concentrations (0.1, 1 and 2 wt%) was incorporated into PCL scaffolds created with a screw-assisted extrusion-based 3D printer with a printing nozzle diameter of 330 µm. The wettability and mechanical properties of the scaffolds were found to be comparable to pure PCL. On the other hand, PANI has significantly higher electrical conductivity, highlighting its potential as an electroactive scaffold. According to the SEM images in Figure 7, the porosity of scaffolds in the 44%–50% range decreases slightly as the PANI loading increases. This phenomenon corresponds to the larger fiber diameter and smaller pore size observed with higher PANI concentrations. Scaffolds must have a high porosity to allow for the diffusion and release of biological substances and nutrients throughout the structure, allowing for optimal cell behavior. The PCL/PANI scaffolds built have an appropriate morphology to allow for nutrient diffusion, cell growth, and migration (Wibowo et al., 2020).

**FIGURE 8:**
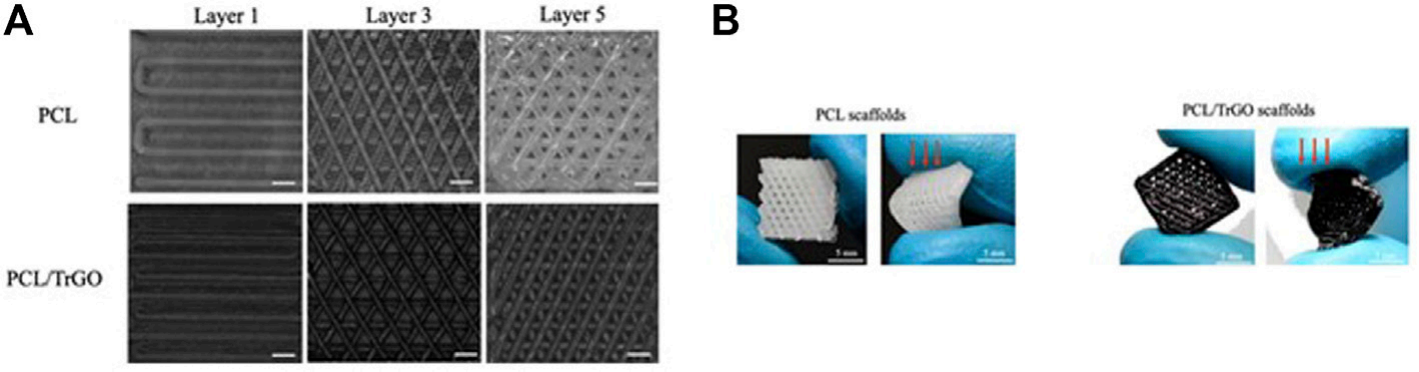
The development of PCL/ TrGO scaffold. **(A)** Images obtained during scaffold printing processes at layers 1, 3, and 5. Scale bar: 300 μm, and **(B)** optical imaging of the frontal scaffold viewpoint (left) and after exerting qualitative stress (right). Images **(A, B)** were adapted from Angulo-pineda et al. (2020) under the Creative Commons Attribution License.

The 3DP technique has also been employed to create novel electroactive polycaprolactone (PCL) scaffolds containing conductive thermally reduced graphene oxide (TrGO) nanoparticles for antibacterial and TE applications (Angulo-pineda et al., 2020). Similarly, the 3DP technique was chosen due to the porous structure’s excellent controllability. Figure 8A shows examples of scaffolds captured by the 3D printer camera after each layer was completed. Based on these images, it was determined that the presence of TrGO nanofiller did not affect the printability or scaffold properties. Meanwhile, Figure 8B shows optical images of the scaffolds after processing, both with and without TrGO filler. The addition of TrGO in the PCL scaffold inhibited bacterial growth to a lesser extent than pure PCL without electrical stimulation (ES). Notably, when the 3D-printed electroactive scaffolds were electrically stimulated, bacterial growth on the scaffold surface was eliminated, whereas pure PCL scaffold retained bacterial adhesion after ES.

### 4.2 Bioprinting

Bioprinting is a broad term that refers to any two-dimensional (2D) or three-dimensional (3D) printing mode that incorporates biological ingredients to create functional tissues and organs. As viable cells are integrated directly into the printing process, it is a revolutionary method from the 3DP technique (Ligon et al., 2017). Furthermore, the concept of bioprinting stems from their ability to print biologically compatible ‘inks’ consist of scaffolds, live cells, growth factors, and other biocompatible materials rather than the plastic and metal inks used in traditional 3DP (Jessop et al., 2017; Athukorala et al., 2021; Langridge et al., 2021).

The 3D bioprinting technology has grown in popularity due to its precise deposition, cost-effectiveness, simplicity, and cell distribution controllability in terms of shape, size, internal porosity, and interconnectivity (Li et al., 2016; Derakhshanfar et al., 2018). Three-dimensional (3D) bioprinting technology advancements have resulted in the emergence of four-dimensional (4D) bioprinting. In brief, it used a variety of stimuli-responsive materials, such as electrical, thermal, humidity, pressure, and photo-responsive materials, to create smart scaffold materials that can transform structurally and respond to internal and external stimuli for post-printing functionality, as well as possess the environmental and structural dynamics of native tissues (Ramburrun et al., 2019).

Spencer et al. (2019) created a complex 3D cell-laden conductive hydrogel composite with a synthesized gelatin methacryloyl (GelMA)/poly (3,4-ethylenedioxythiophene): poly (styrenesulfonate) PEDOT: PSS bio-ink. This work was inspired by their previous study, which revealed that GelMA hydrogel experienced more excellent conductivity value and promoted better viability and spreading of C2C12 cells in 3D as PEDOT: PSS was incorporated into the GelMA system for 0.1% (w/v). Rastin et al. (2020a) also developed a biocompatible bio-ink for current 3D bioprinting by combining methylcellulose and kappa-carrageenan (MC/κCA) hydrogel with PEDOT: PSS conducting polymer. The produced bio-ink displayed a highly thixotropic behavior that may be modified by varying the MC and κCA concentrations to achieve facile printing with great shape fidelity. It was also capable of producing a physiological scale construct without the use of a secondary support bath. Furthermore, the electrical conductivity of the ink was controlled by varying the concentration of PEDOT: PSS (Rastin, et al., 2020b).

In addition, Rastin et al. (2020a) produced a new electroconductive cell-laden bio-ink composed of Ti3C2 MXene nanosheets dispersed homogeneously within a hyaluronic acid/alginate (HA/Alg) hydrogel to overcome the low electrical conductivity of most commercially available bio-inks. Furthermore, the electrical conductivity of the nanocomposite bio-ink with respect to the MXene nanosheets content (1 mg/ml and 5 mg/ml) were significantly higher than the pure hydrogel with electrical conductivity of 1103 ± 93 μS/cm, compared to 5500 ± 85 μS/cm and 7200 ± 126 μS/cm, respectively. Because of the highly thixotropic behavior of the HA/Alg hydrogel, these new hybrid bio-inks have demonstrated outstanding printability with excellent shape retention and resolution. Moreover, the MXene nanocomposite ink demonstrated enhanced mechanical properties in terms of compression strength compared to the pristine HA/Alg hydrogel due to stronger molecular interactions between MXene and hydrophilic polymers which unattainable with PEDOT: PSS conductive inks (Rastin et al., 2020b). These results suggest that MXene bio-inks have a bright future in 3D bioprinting for TE applications.

Yang et al. (2021) recently reported impressive results on the used the 4D printing technique to create a cell-laden fibrous structure. This work was accomplished by using gelatin, known for its swelling properties, to roll up the cell laden GelMA fibers, mimicking the natural structure of skeletal muscle tissues. 4D printing can mimic the dynamics of native tissues, which becomes one of the most significant differences between 3DP and 4D printing. While 3D printed components remain relatively static, 4D printed structures can transform into another shape or configuration when subjected to external stimuli. Consequently, the 4D printed structures possessed enhanced structural and biological functionality. The advantages and disadvantages of these scaffold fabrication methods has been summarized in Table 3.

**TABLE 3:** The advantages and disadvantages of each fabricating method.

Methods	Advantages	Disadvantages	References
Electrospinning	• Scalable	• Instable jetting	Eltom et al. (2019); Mirjalili and Zohoori. (2016); Roshandel and Dorkoosh. (2021); Wang et al. (2021)
	• Cost-effective	• Involves an organic solvent can be toxic	
	• Great porosity scaffold	• Many processing parameters to be considered	
	• Controllable fiber dimension	• Complex process to obtain 3D structures with adequate pore sizes	
	• Long and continuous nanofibers can be produced		
3DP	• Rapid procedure	• Poor mechanical properties	Roshandel and Dorkoosh. (2021); Thiam et al. (2022)
	• Economical and accessible process	• Inability to use wide range of materials	
	• Ability to reproduce native tissue-resembling structure	• Selective process for eliminating trapped powder	
Bioprinting	• Inexpensive	• Depends on the presence of cells • Difficulties in controlling printing quality	Collins et al. (2021); Derakhshanfar et al. (2018); Eltom et al. (2019); Huang et al. (2017)
	• Rapid process		
	• Great structural complexity		
	• Precise deposition and accuracy		
	• Excellent poor interconnectivity		
	• Supports high cell viability of 80–90%		
	• Good porosity and pore size controllability		

## 5 Gold Nanostructures-Based Scaffolds

Over the years, researchers have utilized polymeric and inorganic nanoparticles (NPs), particularly gold nanoparticles (AuNPs) for conductivity enhancement of scaffold for tissue engineering. This is especially important for electroactive tissues, such as cardiac and neuronal to possess enhanced ability to transport electrical signals between cells and to the entire tissues system. AuNPs can be easily tailored to different sizes and shapes, possess size-dependent optical properties, and can be efficiently functionalized (Yadid et al., 2019). As mentioned, AuNPs can be synthesized in a variety of shapes and sizes, which determine their physical properties and suitability for a variety of applications. In tissue engineering, AuNPs are utilized to improve the mechanical characteristics of scaffolds, electrical interaction between cells, cell adhesion, and to promote stem-cell proliferation, differentiation, and maturation. AuNPs are also employed as tissue adhesives, allowing engineered tissue patches to be integrated with native organs.

A study conducted by Baranes et al. (2016) has developed a nanocomposite scaffold composed of PCL/gelatin nanofibers via electrospinning process, which further evaporated with AuNPs. The addition of AuNPs to the fibers offered additional topographical and anchoring sites for improved morphogenesis. Moreover, the neuronal cell line behavior showed more complex neural networks as the neuronal growth became more extended while axon became more elongated. Afterwards, Khan et al. (2018) fabricated a nanofiber-based tubes scaffold comprised of polyvinylpyrrolidone and gold nanoparticles (PVP/AuNPs) *via* electrospinning for neuroscience application. This study revealed that neat PVP possessed limited voltage capacity, which is insufficient for axons application potentially, while PVP/AuNPs tube possessed excellent voltage capacity in requisite range for axon.

Furthermore, Nekounam et al. (2020) has fabricated carbon nanofiber/gold nanoparticles (CNF/AuNPs) conductive scaffold for bone defects repairing. The electrical conductivity of the scaffold enhanced from 2.74 ± 0.02 S/cm to 4.96 ± 0.06 S/cm upon the addition of 2.5% AuNPs. Notably, the LDH proliferation assay revealed significant cell proliferation of Mg-63 cells on CNF/AuNPs scaffold, equivalent to the control sample, up to 72 h. Moreover, a cardiac patch embedded with gold nanowires in collagen fibers by electrospinning has successfully improved myocardial infarction (MI) therapeutics (Tian et al., 2021). Notably, the gold nanowires provided additional mechanical strength and enhanced cell proliferation. Another significant role of the gold nanowires in the collagen fiber matrix was strengthening cell to cell interactions by promoting cellular adhesion and repeatable branching. Hence, the rationale to incorporate metallic nanoparticles for conductivity enhancement are promising, yet there are still some significant impediments to the *in-vivo* deployment of these materials since the quantity of scientific reports is insufficient to allow them to be commercialized in medical practice (Khan et al., 2020). Therefore, current research is shifting toward developing injectable, adhesive, and in situ-curable conductive scaffolds for electrically active tissues, such as cardiac and neuronal tissues (Meyers et al., 2021).

## 6 Conducting Polymers-Based Scaffolds

Conducting polymers (CPs) are organic materials with conjugated p-orbitals which results in electron delocalization and becomes highly conductive (Burnstine-Townley et al., 2020). Most CPs undergoes polymer blending for tissue engineering purposes to form a hybrid conductive scaffold with improved processability enhanced and mechanical properties. In contrast to prior cases, where the conductivity of the scaffold was enhanced by the inclusion of nanostructures, the conductivity of the scaffold in this case is enhanced by the addition of a homogenous polymer blend. However, the CPs in pristine form possess relatively low conductivity value. Hence, doping process of CPs with acids, or polar organic solvents, or ionic liquids are crucial to enhance the electrical conductance by manipulating the surface charge of the scaffold and electrostatic interactions between the scaffold and cells (Burnstine-Townley et al., 2020; Z.; Zhu et al., 2017).

Mawad et al. (2016) conducted intriguing *ex vivo* and *in vivo* tests to demonstrate the efficacy of conductive scaffolds at tissue level. Furthermore, phytic acid as a binding agent has provided good integration of PANI to a chitosan film *via* ionic crosslinking which formed a stable scaffold with protracted conductivity. The conductive patch was implanted into explanted and infarcted rat hearts through a suture-less method, namely photo-adhesion process with a green laser. The PANI patch enhanced cardiac conduction velocity, while the nonconductive control showed no impact. When implanted *in vivo* into healthy rats, the patch was able to attenuate generated arrhythmias, outperforming both sham surgery and nonconductive control. This work demonstrates the immediate medicinal utilization conductive scaffolds for repairing injured cardiac tissue.

Moreover, another effort made by L. Wang et al., 2017a) has shown the effectiveness of conductive scaffolds *in vivo*. Herein, they fabricated an electroactive nanofibrous scaffold consisted of poly (l-lactic acid)/polyaniline (PLA/PANI) *via* electrospinning method. To assess the biocompatibility of PLA/PANI nanofibrous scaffold, a rat cardiomyoblast cell line (H9c2) was first cultivated on these nanofibrous scaffold. Most of the cells on PLA/PANI (1.5) and PLA/PANI 3) displayed green fluorescence after seeding for 24 h, signifying that they were alive. Also, the cell viability and proliferation of these PLA/PANI scaffolds was equivalent to PLA nanofibrous sheets, an FDA approved biomaterial. On top of that, the myogenic differentiation of H9c2 cells on these conductive scaffolds demonstrated by MYH2 immunofluorescence staining visualized a multinucleated fused myotubes with high ordered structure. This work reveals the potential of PLA/PANI scaffold for CTE application as it promotes differentiation of H9c2 cells in terms of myotube quantity, myotube length, maturation index, and fusion index. Furthermore, the primary cardiomyocytes (CMs) augmented onto the PLA/PANI scaffolds showed majority of the cells are alive and vibrant after 36 h. Also, CMs on PLA/PANI scaffolds expressed significantly more F-actin fibers across all geometrical aspects while CMs on pure PLA sheets demonstrated little F-actin fiber expression and a rectangular morphology. Notably, elongated CMs with well-defined stress fibers were seen on PLA/PANI (1.5) and PLA/PANI 3) nanofibrous sheets but not on PLA. Additionally, the CMs on PLA/PANI 3) sheets were interconnected and spatially oriented which led to cell-cell interactions enhancement as revealed by a fast Fourier transform (FFT) analysis. All of these findings were enhanced by the increment of PANI contents in the scaffold system which might be attributed to the beneficial effect of conductivity which enhance cellular spreading and alignment and cell-cell interactions. Remarkably, CMs on PLA/PANI conductive nanofibrous sheets continued to beat spontaneously with regular contraction patterns for 21 days, demonstrating tremendous potentiality in clinical interventions for CTE.

A study conducted by Roshanbinfar et al. (2018) eliminated the risk of cardiac arrhythmia caused by poor electrical coupling by developing a biohybrid hydrogel composed of collagen, alginate, and PEDOT: PSS (referred to simply as “eCA-gels”). Herein, the incorporation of PEDOT: PSS in the hydrogel improves electrical coupling within the graft and significantly improves the beating frequencies, up to 200 beats min^−1^, resulting in the highest endogenous beating frequency of engineered cardiac tissue described to date. Furthermore, the inclusion of PEDOT: PSS improved surface coverage of CMs substantially. Also, CMs made more cell-to-cell connections and had better sarcomeric striations, which translates to a higher number of aligned myofibrils. These findings suggest that eCA-gels promote cellular alignment, elongation, and linear orientation while increasing intercellular electrical coupling in CMs.

In an effort to investigate the effect of conductive scaffold towards neural tissue engineering, Sadeghi et al. (2018) fabricated PCL/Chitosan/Polypyrrole (PPy) nanofibrous composite scaffold *via* electrospinning process. Notably, cell proliferation rate of PC12 cell line on the PCL/PPy scaffolds showed 2.75 x increment due to the scaffold electrically charged surface. Also, the neuronal branching from PC12 cells implanted on the surface of the PCL/PPy scaffold was clearly apparent. Similarly, the PCL/Chitosan/PPy scaffold may enhance neuron-like PC12 cell attachment and boost cell spreading and proliferation of PC12 cells. Integrating PPy to the polymeric composite blending enhanced the development of PC12 neural-like cells by 52%, as a result from the electroactivity of the nanofibrous scaffold. Particularly, this work demonstrated the ability of PCL/Chitosan/PPy nanofibrous scaffolds to aid the growth and proliferation of PC12 cells.

## 7 Conclusion and Future Prospects

This review highlights some recent advancements in the fabrication of electroactive conductive scaffolds in TE applications. In general, different techniques have been used, which are classified as conventional and rapid prototyping. Based on the research methodology, electrospinning, 3D printing, and bioprinting have generated much interest in developing conductive scaffolds with the desired properties. As they provide nano-to micrometer fiber diameter, the electrospinning technique is classified as a conventional method with the best ability to mimic the extracellular matrix (ECM) of native tissue. However, the electrospinning technique faces some challenges, such as a random porous structure with limited reproducibility and a lack of control over the size, geometry, and spatial distribution of pores (Yuan et al., 2017). As a result, 3D printing and bioprinting techniques emerge as innovation continues to accelerate with the motivation to overcome the limitations of traditional methods. Ideally, conductive materials as ink or bio-ink to serve electrical conductivity properties are highly desirable. As a result of incorporating conductive materials, the barrier of current polymeric ink with a poor electrical conductivity mismatched with the native tissue environment will be reduced. Furthermore, the use of conductive materials as ink or bio-ink has aided in creating anatomical-size structures with high form accuracy and resolution. Higher resolution for bioprinting is always a concern because it necessitates higher shear pressures, which reduce cell viability (Saska et al., 2021). As one of the developing technologies, 4D printing and its contribution is also briefly highlighted. The fabrication technique used is essentially determined by the application, type of tissues, and desired morphology/geometry of the electroactive scaffold and prints. All the reported methods, on the other hand, have a 100% chance of producing fully functional organs. The future of these electroactive scaffold-based conducting polymers appear bright as conductivity is one of the important features required for developing scaffolds specifically targeted for electroactive tissues. However, the incorporation of conducting polymers into scaffold construct *via* 3D printing and bioprinting require further research and exploration to be established into tissue engineering application. For instance, high resolution bio-printed scaffolds tend to retain low cell viability due the high sheer pressure. Hence, by integrating an optimum concentration of CPs would be favorable for high cell viability scaffold system (Rastin et al., 2020a). Moreover, future development of these electroactive scaffold by integrating nanomaterials into the 3D network scaffold construct would be significantly improve electrical interaction, cell adhesion and proliferation, and mechanical characteristics. Eventually, an extensive study on revealing the stability of the electroactive scaffolds must be conducted to ensure excellent cell adhesion and mechanical stability at the injured tissues. The scaffold must preserve structural integrity and stability while being implanted into the defect site, and provide adequate biomechanical support during tissue regeneration and structural degradation processes (Venugopal et al., 2008). More advances in tissue engineering and regenerative medicine will lead to broad commercialization and application of the electroactive scaffold-based conducting polymers which significantly enrich patients’ quality of life.
