# Supplementary material for: Fabrication Methods of Electroactive Scaffold-Based Conducting Polymers for Tissue Engineering Application: A Review
Source: Front Bioeng Biotechnol. 2022 Jul 7;10:876696. doi: 10.3389/fbioe.2022.876696 (PMC9300926; doi:10.3389/fbioe.2022.876696)
Supplement: Supplementary file 1 [file Table1.docx]

**Table S1. Summary of the scaffold fabrication methods.**

| ****Methods**** | ****Scaffold Composition**** | ****Parameters/ Major findings**** | ****Conductivity (S/m)**** | ****Application**** | ****Reference**** |
| --- | --- | --- | --- | --- | --- |
| **Electrospinning** | **Chitosan/ PEDOT: PSS** | **Flow rate: 0.5 ml/h**  **Speed of rotating drum collector: 2500 rpm Applied voltage: 20 kV**  **Nozzle-to-collector distance: 18 cm** | **7.63 × 10^-3^** | **Cardiac tissue engineering** | **(Abedi et al., 2019)** |
|  | **PVA/ PEDOT: PSS** | **Flow rate: 0.4 ml/h**  **Speed of rotating drum collector: 1000 rpm Applied voltage: 25 kV**  **Needle-to-drum distance: 18 cm** | **2.00 × 10^-3^** | **Neural tissue engineering** | **(Babaie et al., 2020)** |
|  | **PEDOT: PSS/ silk** | **Flow rate: 0.8 ml/h**  **Speed of rotating drum collector: 0 rpm (static)**  **Applied voltage: 15 kV**  **Tip-to-collector distance: 10 cm Relative humidity: 25%**  **Needle gauge size: 19 G** | **N/A** | **Neural tissue engineering** | **(Magaz et al., 2020)** |
|  | **PCL/ Gelatin/ AuNPs** | - **The addition of AuNPs to the fibers offered additional topographical and anchoring sites for improved morphogenesis.** - **Neuronal growth became more extended while axon became more elongated.** | **N/A** | **Neural tissue engineering** | **(Baranes et al., 2016)** |
|  | **PVP/ AuNPs** | - **The neat PVP possessed very small potential voltage capacity.** - **PVP/AuNPs tube possessed good potential voltage, which is in the range required for axon from –75mV to +75 mV neuro signal.** | **N/A** | **Neural tissue engineering** | **(M. Q. Khan et al., 2018)** |
|  | **CNF/ AuNPs** | - **The electrical conductivity of the scaffold enhanced from 2.74±0.02 S/cm to 4.96±0.06 S/cm upon the addition of 2.5% AuNPs.** - **The LDH proliferation assay revealed significant cell proliferation of Mg-63 cells on CNF/ AuNPs scaffold, equivalent to the control sample.** | **496±0.06** | **Bone tissue engineering** | **(Nekounam et al., 2020)** |
|  | **Collagen/ AuNW** | - **The AuNW provided additional mechanical strength and enhanced cell proliferation.** - **AuNW in the collagen fiber matrix strengthened cell to cell interactions by promoting cellular adhesion and repeatable branching.** | **N/A** | **Cardiac tissue engineering** | **(Tian et al., 2021)** |
| **3DP** | **HA/ PVOH** | - **The flowability of the precursor powders improved as the HA content decreases, as indicated by the reduction of repose angle and the flow time.** - **Consequently, these phenomenon improve the printability process.** - **Materials that possessed lower angle of repose and faster flow time are easily being printed.** - **50HA:50PVOH (wt.%) precursor powder was shown to be the optimum ratio in developing a stable printing construct with no damage observed.** - **In addition, 50 wt.% HA green scaffolds possessed excellent bonding between layers as no structural damage and de-bonding of layers arise during the de-powdering and handling process.** - **The 50 wt.% HA scaffolds printed along the Y-axis exhibited higher yield and ultimate compressive strength (0.88 ± 0.02 MPa) compared to X-axis (0.76 ± 0.02MPa) due to the isotropic nature of the 3D printed parts.** - **The ultimate compressive strength value along Y-axis was categorized within the lower range reported for cancellous bone and deemed to be mechanically suitable for the intended applications; bone tissue engineering, with an average total porosity of 55.1 ± 0.9%.** | **N/A** | **Bone tissue engineering** | **(Cox et al., 2015)** |
|  | **PCL/ PANI** | - **The morphological evaluation *via* SEM micrograph of the PCL/ PANI scaffolds at all PANI concentration revealed a regular geometry with uniform pore size and interconnected circular cross-sections.** - **Moreover, the scaffold porosity reduced with an increasing PANI concentration due to the increased in fiber diameter.** - **In terms of the mechanical evaluation, the compressive strength of the scaffolds improved with the increment of PANI concentration.** - **Moreover, the compressive strength values obtained falls in the range for cancellous bone applications.** - **Similarly, the inclusion of PANI enhanced the conductivity of the scaffolds which lies within the conductivity region of cancellous and cortical bone.** - **At higher concentrations of PANI (1 and 2% wt.), the biological assessment demonstrated a cytotoxic effect for the scaffold cytocompatibility, while 0.1% wt. of PANI demonstrated good cytocompatibility yet conductive.** | **2.46 × 10^-2^** | **Bone tissue engineering** | **(Wibowo et al., 2020)** |
|  | **PCL/ TrGO** | **The PCL/ TrGO composites were synthesized *via* a melt mixing process. The electrical conductivity of the composite films (2D) after the melt mixing process was lower by one magnitude, compared to their electrical properties after the 3D-printing process. The difference in conductivity values was caused by the melting process involved to construct the scaffold.** | **6.80 × 10^-5^** | **Antibacterial and tissue engineering** | **(Angulo-pineda et al., 2020)** |
| **Bioprinting** | **GelMA/ PEDOT: PSS** | - **Initially, the PEDOT: PSS underwent a modification *via* cross-linking process with bivalent calcium ions and subsequently went for a secondary photopolymerization step with a visible light to cross-link the GelMA component.** - **The mechanical stiffness of the GelMA/ PEDOT: PSS scaffolds improved with the addition of the salt solution.** - **However, the conductivity of the composite scaffolds showed insignificant changes as the concentration of calcium chloride increases.** - **This scenario opposed to the previous findings that revealed conductivity enhancement of PEDOT: PSS hydrogel with the presence of bivalent Mg^2+^ and Ca^2+^.** - **The possibilities of these occurrence might cause by the diffuse out of Ca^2+^ ions from the hydrogel system or transformation from bivalent to monovalent ion as the hydrogel was rinsed with DI water prior to the impedance testing.** | **N/A** | **Biomedical application** | **(Spencer et al., 2019)** |
|  | **MC/ κCA/ PEDOT: PSS** | - **At constant concentration of κCA while increase in MC concentration, the MC/ κCA hydrogels displayed a reduction in swelling ratio due to denser polymer entanglement.** - **Similarly, at higher concentration of κCA, the swelling ratio reduced due to the increased formation of double helix structure.** - **In addition, the MC/ κCA hydrogels demonstrated a gradual increase of compressive modulus upon increasing the MC concentration as more hydrophobic interaction of MC chains were formed.** - **Also, a stronger hydrogels were fabricated at higher concentration of κCA, since more ionic interaction occurred between K^+^ and κCA chains.** - **Moreover, the incorporation of κCA has improved the electrical conductivity of pure MC due to the ionic nature of κCA hydrogel.** - **The conductivity studies revealed that the electrical conductivity of the hydrogel showed a progressive rise as the PEDOT: PSS content in the system increases.** - **Previously, there was a finding asserted that the cell viability declined with the addition of PEDOT: PSS above 0.1 wt.% concentration.** - **Herein, the MC-8/κCA-1/PEDOT:PSS-0.1 hydrogel demonstrated high cell viability (>96%) over a week in both bulk (unprinted ink) and 3D bio-printed structure.** | **1.21 × 10^-1^** | **Tissue engineering** | **(Rastin et al., 2020)** |
|  | **Ti_3_C_2_ MXene/ HA/ Alg** | - **The electrical conductivity of the inks showed an improvement upon the addition of 1 and 5 mg/ml of Ti_3_C_2_ MXene nanosheets into the HA/Alg system, due to the inherent electrical conductivity properties of MXene.** - **Hydrogel sample with 5 mg/ml concentration of MXene demonstrated enhanced compressive modulus compared to the pristine HA/Alg hydrogel.** - **In contrast, the sample with 1 mg/ml concentration of MXene showed insignificant effect on the compressive modulus.** - **Moreover, the swelling ratio of the HA/Alg hydrogel upon the addition of 5 mg/ml concentration of MXene reduced, as a result of greater molecular interactions between MXene and hydrophilic polymers.** - **Herein, the 1 mg/ml MXene nanosheets were chosen as the optimal concentration for 3D bioprinting since they provided higher cell growth indicated from the intensity of green fluorescence amplified over time.** | **7.20 × 10^-1^** | **Neural tissue engineering** | **(Rastin et al., 2020)** |
|  | **C2C12-laden GelMA** | **Electric field intensity: 0.8 kV cm^-1^ Applying time: 12s**  **Cell number: 15 × 106 cells mL^-1^** | **N/A** | **Tissue engineering** | **(Yang et al., 2021)** |
